# Epidemiology and Association Rules Analysis for Pulmonary Tuberculosis Cases with Extrapulmonary Tuberculosis from Age and Gender Perspective: A Large-Scale Retrospective Multicenter Observational Study in China

**DOI:** 10.1155/2023/5562495

**Published:** 2023-07-31

**Authors:** Wanli Kang, Jiajia Yu, Chen Liang, Quanhong Wang, Liang Li, Jian Du, Hongyan Chen, Jianxiong Liu, Jinshan Ma, Mingwu Li, Jingmin Qin, Wei Shu, Peilan Zong, Yi Zhang, Xiaofeng Yan, Zhiyi Yang, Zaoxian Mei, Qunyi Deng, Pu Wang, Wenge Han, Meiying Wu, Ling Chen, Xinguo Zhao, Lei Tan, Fujian Li, Chao Zheng, Hongwei Liu, Xinjie Li, Ertai A., Yingrong Du, Fenglin Liu, Wenyu Cui, Song Yang, Xiaohong Chen, Junfeng Han, Qingyao Xie, Yanmei Feng, Wenyu Liu, Peijun Tang, Jianyong Zhang, Jian Zheng, Dawei Chen, Xiangyang Yao, Tong Ren, Yan Li, Yuanyuan Li, Lei Wu, Qiang Song, Mei Yang, Jian Zhang, Yuanyuan Liu, Shuliang Guo, Kun Yan, Xinghua Shen, Dan Lei, Yanli Zhang, Youcai Li, Yongkang Dong, Shenjie Tang

**Affiliations:** ^1^Beijing Chest Hospital, Capital Medical University, Beijing Tuberculosis and Thoracic Tumor Research Institute, Beijing 101149, China; ^2^Taiyuan Fourth People's Hospital, Number 231, Xikuang Street, Wanbailin District, Taiyuan, Shanxi 030024, China; ^3^Shenyang Chest Hospital, No. 11 Beihai Street, Dadong District, Shenyang110044, China; ^4^Guang Zhou Chest Hospital, No. 62, Heng Zhi Gang Road, Yuexiu District, Guangzhou, Guangdong 510095, China; ^5^Chest Hospital of Xinjiang, No. 106, Yan ‘An Road, Tianshan District, Urumqi, Xinjiang 830049, China; ^6^The Third People's Hospital of Kunming, No. 319 Wu Jing Road, Kunming, Yunnan 650041, China; ^7^Shandong Provincial Chest Hospital, No. 12, Lieshishandong Road, Licheng District, Jinan, Shandong 250000, China; ^8^Jiangxi Chest (Third People) Hospital, No. 346 Dieshan Road, Donghu District, Nanchang, Jiangxi 330006, China; ^9^Chang Chun Infectious Diseases Hospital, No. 2699, Sandao Section, Changji South Line, Erdao District, Changchun, Jilin 130123, China; ^10^Chongqing Public Health Medical Center, No. 109, Baoyu Road, Geleshan Town, Shapingba District, Chongqing 400036, China; ^11^Fuzhou Pulmonary Hospital of Fujian, No. 2, Lakeside, Cangshan District, Fuzhou 350008, China; ^12^Tianjin Haihe Hospital, Number 890, Shuanggangzhenjingu Road, Jinnan District, Tianjin 300350, China; ^13^Third People's Hospital of Shenzhen, 29 Bulan Road, District Longgang, Shenzhen 518112, China; ^14^The First Affiliated Hospital of Chongqing Medical University, No. 1 Youyi Road, Yuzhong District, Chongqing 400016, China; ^15^Weifang No. 2 People's Hospital, No. 7th Yuanxiao Street, Kuiwen District 261041, China; ^16^The Fifth People's Hospital of Suzhou, No. 10 Guangqian Road, Suzhou, Jiangsu 215000, China; ^17^Affiliated Hospital of Zunyi Medical College, No. 149 Delian Road, Zunyi, Guizhou 563000, China; ^18^The Fifth People's Hospital of Wuxi, No. 1215, GuangRui Road, Wuxi 214001, China; ^19^TB Hospital of Siping City, No. 10 Dongshan Road, Tiedong District, Siping, Jilin Province 136001, China; ^20^Baoding Hospital for Infectious Disease, No. 608 Dongfeng East Road, Lianchi District, Baoding, Hebei 071000, China; ^21^The First Affiliated of XiaMen University, ZhenhaiRoud, Siming District, Xiamen, Fujian, China

## Abstract

**Background:**

Tuberculosis (TB), a multisystemic disease with protean presentation, remains a major global health problem. Although concurrent pulmonary tuberculosis (PTB) and extrapulmonary tuberculosis (EPTB) cases are commonly observed clinically, knowledge regarding concurrent PTB-EPTB is limited. Here, a large-scale multicenter observational study conducted in China aimed to study the epidemiology of concurrent PTB-EPTB cases by diagnostically defining TB types and then implementing association rules analysis.

**Methods:**

The retrospective study was conducted at 21 hospitals in 15 provinces in China and included all inpatients with confirmed TB diagnoses admitted from Jan 2011 to Dec 2017. Association rules analysis was conducted for cases with concurrent PTB and various types of EPTB using the Apriori algorithm.

**Results:**

Evaluation of 438,979TB inpatients indicated PTB was the most commonly diagnosed (82.05%) followed by tuberculous pleurisy (23.62%). Concurrent PTB-EPTB was found in 129,422 cases (29.48%) of which tuberculous pleurisy was the most common concurrent EPTB type observed. The multivariable logistic regression models demonstrated that odds ratios of concurrent PTB-EPTB cases varied by gender and age group. For PTB cases with concurrent EPTB, the strongest association was found between PTB and concurrent bronchial tuberculosis (lift = 1.09). For EPTB cases with concurrent PTB, the strongest association was found between pharyngeal/laryngeal tuberculosis and concurrent PTB (lift = 1.11). Confidence and lift values of concurrent PTB-EPTB cases varied with gender and age.

**Conclusions:**

Numerous concurrent PTB-EPTB case types were observed, with confidence and lift values varying with gender and age. Clinicians should screen for concurrent PTB-EPTB in order to improve treatment outcomes.

## 1. Background

Tuberculosis (TB) is an infectious disease caused by the bacillus Mycobacterium tuberculosis. Despite eradication efforts, TB remains a major global health problem and a cause of serious illness for millions of people each year. According to the World Health Organization (WHO), the estimated global incidence of TB was approximately 10.6 million cases in 2021. [1]. TB typically affects the lungs (pulmonary TB, PTB) but can also affect other sites (extrapulmonary TB, EPTB) that include pleura, lymph nodes, abdomen, genitourinary tract, skin, joints and bones, and meninges [2–4].

In recent years, considerable effort has been expended to gain a deeper understanding of TB [5–7], a multisystemic disease with a protean presentation. In fact, in clinical practice, PTB and EPTB have been both detected in the same patient [8, 9]. It was reported that about 10%–50% of EPTB patients have concomitant pulmonary involvement [8]. The effective treatment regimens for concurrent PTB-EPTB cases were shown to be more difficult to administer and different from effective regimens used for treating single PTB or EPTB cases [10]. As information pertaining to concurrent PTB-EPTB is scarce, research efforts are needed to diagnostically define TB case types and explore the epidemiology and association rules between PTB and different types of concurrent EPTB. In this work, we addressed this gap in knowledge by conducting a large-scale multicenter observational study. The resulting acquired knowledge will be used to alert clinicians to the common occurrence of concurrent PTB-EPTB disease and improve treatment regimens used for such cases.

## 2. Methods

### 2.1. Study Subjects

The retrospective study was performed at 21 Hospitals in 15 provinces in China. All inpatients with confirmed TB diagnoses in medical databases from Jan 2011 to Dec 2017 were included in the study. TB was mainly categorized by lesion site. Diagnosis of TB was made based on WHO guidelines [11] and the Clinical Diagnosis Standard of TB issued by the Chinese Medical Association [12]. In general, TB was diagnosed using both traditional and modern methods based on clinical symptoms and physical signs together with results obtained using bacteriological methods (including sputum smear microscopy, bacterial culture, and molecular diagnostic methods), tuberculin skin testing (TST) or purified protein derivative (PPD) testing, T-SPOT. TB, X-ray findings, CT scan, ultrasound, bronchoscopy, and outcomes of treatment with a course of antituberculosis chemotherapy. Based on clinical manifestations and abnormal chest radiographs, all PTB patients should meet one of the following criteria: (1) sputum acid-fast bacilli (AFB) smear or culture positive; (2) molecular-based assays (GeneXpert MTB/RIF or line probe assays) positive; (3) a pathological diagnosis of TB in lung specimens. Bronchial tuberculosis is diagnosed on histopathological examination of bronchoscopically obtained specimens showing granulomatous inflammation with caseation necrosis and/or positive AFB, culture, and molecular-based assays on the microbiological examination. EPTB can potentially affect any organ in the body and may present itself with an extremely wide spectrum of signs and symptoms depending on the organ affected, the aggressiveness of the disease, and the immune response of the host. Based on clinical manifestations and abnormal imaging and laboratory results, all EPTB patients met at least one of the following criteria: one culture-positive specimen taken from the lesion site; one culture-positive fluid sample (such as ascitic or pleural fluid or cerebrospinal fluid) from the lesion site; fluid samples with positive GeneXpert MTB/RIF assay results; histopathological changes in the lesion site that supported a diagnosis of EPTB; and strong clinical confirmation of active EPTB and positive response to anti-TB treatment. TB cases in the study may be bacteriologically confirmed or clinically diagnosed TB cases such as smear-negative TB.

### 2.2. Data Management and Statistical Analysis

Measures taken to guarantee data quality included both the implementation of a standardized study protocol and standardized training of research staff. Moreover, trained health workers collected medical information using a standardized questionnaire. Meanwhile, we obtained clinical characteristics of TB-afflicted inpatients (e.g., age, gender, site of disease) from medical records. Descriptive statistical analysis included frequencies and proportions with 95% confidence intervals (CIs) for categorical variables. Multivariable logistic regression analysis was used to examine the associations of gender and age group, and determinations of odds ratios were conducted for concurrent PTB-EPTB cases. *P* < 0.05 was the threshold for statistical significance.

Association rules analysis, a technique used to discover relationships hidden in large databases, was used in this work. This technique, which was developed for the computer sciences, has been used in a variety of other fields [13–15]. The Apriori algorithm makes it possible to apply a set of association rules to data mining. The principle of Apriori is based on two steps: the first step searches for item sets that exceed the minimum support threshold value, while the second step generates association rules and then filters them by selecting “confidence” item sets (based on a threshold) from item sets found in the first step [16, 17]. For the association rule of *A* concurrent with *B*, support, confidence, and lift were defined as follows: support = *P*(*A*), confidence = *P*(*B*|*A*), lift = *P*(*A*∩*B*)/[*P*(*A*)∗*P*(*B*)] whereby *A* is antecedent and *B* is consequent. Lift was used to evaluate the magnitude of association rules whereby lift >1 indicates a positive association rule. Association rules for concurrent PTB and diverse types of EPTB were analyzed using the Apriori algorithm by setting the minimum support degree and the minimum confidence degree.

All data were collected in MS Office Excel (Microsoft, Redmond, WA, USA) datasheets, and all analyses were conducted using SPSS software for Windows version 13 (Chicago, USA) and SPSS Modeler version 14.1(IBM Corp, Armonk, NY, USA).

## 3. Results

### 3.1. TB Patient Characteristics

A total of 438,979TB inpatients were included from Jan 2011 to Dec 2017 at 21 hospitals from 15 provinces in China, most of which were specialized tuberculosis hospitals. The ratio of male to female patients was 1.83. The proportion of age ≥ 65 years was the most. In these patients, 83 tuberculous lesion types were detected at 604,114 sites, with each patient harboring, on average, 1.38TB lesion types. The most common types of TB were PTB (82.05%, 95% CI: 81.94%–82.16%), followed by tuberculous pleurisy (23.62%, 95% CI: 23.49%–23.74%) and bronchial tuberculosis (7.01%, 95% CI: 6.94%–7.09%). Types of TB found in proportions of ≥1.0% of total cases are shown in Table 1.

### 3.2. Patients with Concurrent PTB-EPTB

A total of 129,422(29.48%) concurrent PTB-EPTB inpatients were found within the entire group of 438,979 TB inpatients. An age-gender percent-bar-graph of concurrent PTB-EPTB is shown in Figure S1 in the Supplementary Appendix. In <15, 15–24, 25–34, 35–44, 45–55 years age group, the percents of concurrent PTB-EPTB female cases were greater than the corresponding percents of male cases. In the multivariable logistic regression models, patients who were female (OR = 1.119, 95% CI: 1.104–1.134), <15 years (OR = 1.602, 95% CI: 1.521–1.686), 15–24 years (OR = 1.831, 95% CI: 1.792–1.871), 25–34 years (OR = 1.700, 95% CI: 1.664–1.738), 35–44 years (OR = 1.358, 1.327–1.391), and 45–54 years (OR = 1.084, 95% CI: 1.059–1.109) were more likely to have concurrent PTB-EPTB, while those who were aged 55–64 years (OR = 0.971, 95% CI: 0.949–0.994) were less likely to have concurrent PTB-EPTB (Table 2).

### 3.3. The Most Common Concurrent PTB-EPTB Types

According to association rules analysis of concurrent PTB-EPTB cases, the 20 most common concurrent PTB-EPTB case types are listed in Table S1 in the Supplementary Appendix, sorted by case number. Case numbers of concurrent PTB and tuberculous pleurisy (15.35%, 95% CI: 15.25%–15.46%) and concurrent PTB and bronchial tuberculosis (6.28%, 95% CI: 6.20%–6.35%) were greater than numbers of other types of concurrent PTB-EPTB cases.

### 3.4. Association Rules Analysis of Concurrent PTB-EPTB Cases

In order to find most of the possible association rules for antecedent = PTB, the minimum confidence degree was set to 1.00%. After executing the association model, six association rules were obtained. The association rules are shown in Table 3 and were sorted by confidence. The first rule row (ID = 1) in Table 3 was interpreted as demonstrating that for a total of 360,187 PTB cases (instances), PTB accounted for 82.05% of all TB cases (support), while PTB with concurrent tuberculous pleurisy accounted for 18.71% of PTB cases (confidence). The confidence value for concurrent bronchial tuberculosis in PTB cases was next highest (7.65%), followed by tuberculous meningitis (2.72%). The strongest association rule for PTB with concurrent EPTB was found for PTB with concurrent bronchial tuberculosis (lift = 1.09), whereby the lift value of 1.09 indicated that PTB was positively associated with bronchial tuberculosis.

In order to find most of the possible association rules for consequent = PTB, the minimum support degree was set to 0.1%, and the minimum confidence degree was set to 40%. After executing the association model, 22 association rules were obtained, including five rules with confidence values above 70%. The association rules shown in Table 4 are sorted by confidence. The first rule row (ID = 1) in Table 4 was interpreted as showing that 2,382 cases (instances) of pharyngeal/laryngeal tuberculosis accounted for 0.54% of all TB cases (support), while pharyngeal/laryngeal tuberculosis with concurrent PTB accounted for 91.23% of pharyngeal/laryngeal tuberculosis cases (confidence). The confidence value of concurrent PTB in bronchial tuberculosis cases was next highest (89.51%), followed by that of tuberculosis of mediastinal lymph nodes (77.57%). The strongest association rule for EPTB with concurrent PTB was found for pharyngeal/laryngeal tuberculosis (lift = 1.11), indicating that pharyngeal/laryngeal tuberculosis was positively associated with PTB.

### 3.5. Association Rules of Concurrent PTB-EPTB Types with Gender

Most types of TB can be found in both males and females, with obvious exceptions of ovarian tuberculosis, oviduct tuberculosis, etc. We found association rules in males and females by setting the minimum support degree and the minimum confidence degree (Tables S2 and S3 in the Supplementary Appendix). In males, tuberculous empyema with concurrent PTB was the strongest association rule (lift = 1.20), followed by coastal tuberculosis with concurrent PTB (lift = 1.16). In females, bronchial tuberculosis with concurrent PTB was the strongest association rule (lift = 1.64), followed by supraclavicular lymph node tuberculosis with concurrent PTB (lift = 1.56).

### 3.6. Association Rules of Concurrent PTB-EPTB Types with Age

We found association rules in all age groups by setting the minimum support degree and the minimum confidence degree (Tables S4∼S10 in the Supplementary Appendix). In patients <15 years of age, tuberculous meningitis with concurrent PTB was the strongest association rule (lift = 3.89), followed by tuberculosis of axillary lymph nodes with concurrent PTB (lift = 3.78) and cervical vertebra tuberculosis with concurrent PTB (lift = 3.61). In patients aged 15–24 years, splenic tuberculosis with concurrent PTB was the strongest association rule (lift = 2.23), followed by tuberculous myelitis with concurrent PTB (lift = 2.18). In patients aged 25–34, oviduct tuberculosis with concurrent PTB was the strongest association rule (lift = 3.09), followed by endometrial tuberculosis with concurrent PTB (lift = 2.17). In patients aged 35–44 years, the strongest association rule was again oviduct tuberculosis with concurrent PTB (lift = 1.71), with endometrial tuberculosis with concurrent PTB (lift = 1.69) ranked next. In patients aged 45–54 years, the strongest association rule was vocal cord tuberculosis with concurrent PTB (lift = 1.61) that was followed by wrist joint tuberculosis with concurrent PTB (lift = 1.57). In TB patients aged 55–64 years, the strongest association rule was ankle joint tuberculosis with concurrent PTB (lift = 1.55), followed by adrenal tuberculosis with concurrent PTB (lift = 1.51). In TB patients aged ≥ 65 years, the strongest association rule was tuberculous pericarditis with concurrent PTB (lift = 1.42), with hilar lymph nodes with concurrent PTB (lift = 1.30) ranked next.

## 4. Discussion

TB is transmitted when people afflicted with PTB expel M. Tuberculosis bacteria into the air; these airborne bacilli are subsequently breathed in by another person and then settle in the lungs and grow. From there, they can be disseminated throughout the lymphatic or hematogenous systems and subsequently infect single or multiple extrapulmonary sites, such as the pleura, lymph nodes, meninges, bones, and joints. Although PTB is the most common presentation of TB, EPTB contributes considerably to morbidity, lifelong sequelae, and mortality [18]. The mechanisms for EPTB dissemination are complicated [19]. Notably, cases of PTB concurrent with EPTB are common in clinical practice, but data regarding such cases are limited. Given the variety of clinical presentations and the nonspecific systemic symptoms of TB, a more profound understanding of the site distribution of TB is needed. In this study, we delineated the diagnostic types of TB observed in clinical practice and explored the association rules of concurrent PTB-EPTB cases from age and gender perspectives in order to alert clinicians to the frequency of concurrent PTB-EPTB cases within a large TB sample population.

Tuberculous pleurisy is one of the most common forms of EPTB [20]. Here, we found that tuberculous pleurisy (23.62%), thought to primarily result from a hypersensitivity reaction to tuberculous protein [21], was the second most common type of TB observed. Previous studies had also noted concurrent PTB-EPTB cases [8, 9], as Boonsarngsuk et al. [8] demonstrated that 12.2% of TB cases had concurrent PTB-EPTB (120/986). In this study, concurrent PTB-EPTB was found in about 30% of TB patients. We also found that the strongest association rule of PTB concurrent with EPTB was found for PTB concurrence with bronchial tuberculosis (lift = 1.09); indeed, here, 7.65% of PTB cases had concurrent bronchial tuberculosis. This association may stem from the fact that bronchi are adjacent to the lungs, and this close proximity predisposes PTB patients to bronchial tuberculosis. However, this outcome is not inevitable as the proportion of EPTB cases with concurrent PTB varied depending on whether patients were primarily viewed as EPTB or PTB patients. Meanwhile, pharyngeal/laryngeal tuberculosis was an infrequent manifestation of EPTB and was usually seen as a complication of PTB [22]. The strongest association rule of EPTB concurrent with PTB was pharyngeal/laryngeal tuberculosis concurrent with PTB (lift = 1.11). In this study, 91.23% of pharyngeal/laryngeal tuberculosis patients had concurrent PTB, reflecting the fact that expulsion of M. tuberculosis by PTB patients would likely affect the larynx. Consistent with this supposition, most pharyngeal/laryngeal tuberculosis patients had concurrent PTB. These findings suggest that once PTB was diagnosed, patients should be evaluated for bronchial tuberculosis first. Conversely, once a diagnosis of pharyngeal/laryngeal tuberculosis or bronchial tuberculosis is confirmed, attention should be paid to evaluating the patient for PTB. In essence, the diagnosis of TB must be carefully undertaken to avoid misdiagnosis.

Although most types of tuberculous lesions can be found both in males and females, females (OR = 1.119, 95% CI: 1.104–1.134) were more likely to have concurrent PTB-EPTB than males in this study. Jung et al. [23] also found the female gender to be an independent predictor of concomitant endobronchial TB in patients with active PTB (OR = 4.35, 95% CI: 1.78–10.63). Meanwhile, the magnitude of association rules of concurrent PTB-EPTB also varied with gender. For males in this study, tuberculous empyema with concurrent PTB was the strongest association rule (lift = 1.20), with proportions of tuberculous empyema with concurrent PTB in males observed that exceeded 70%. In females, bronchial tuberculosis with concurrent PTB was the strongest association rule (lift = 1.64), with a proportion of bronchial tuberculosis with concurrent PTB in females observed that was greater than 55% although the difference of association rules was not clear, warranting additional investigation.

TB affects all age groups, but the overall best estimate for 2019 revealed that about 90% of cases were adults (aged ≥ 15 years), while TB prevalence has been shown to be strongly associated with age [1]. Here, we found that the strongest association rule in children and adolescents (<15 years) was PTB with concurrent tuberculous meningitis (lift = 3.89), a result that may reflect particular physiological characteristics and immunological mechanisms unique to children and adolescents. We also found that the strongest association rule in TB patients aged 15–24 years was splenic tuberculosis with concurrent PTB (lift = 2.23). Splenic tuberculosis is mainly caused by the hematogenous dissemination of a small number of bacteria that directly spread to the spleen via lymphatic pathways and adjacent organs [24]. Meanwhile, our results showed that oviduct tuberculosis with concurrent PTB was the strongest association rule in groups of patients aged 25–34 years (lift = 3.09) and 35–44 years (lift = 1.71). Indeed, oviduct tuberculosis is an important chronic pelvic disease and an etiological factor underlying infertility. Transmission of infection usually occurs via hematogenous systems, while direct spread from abdominal organs and the peritoneum are also possible [25]. Other EPTB types including isolated vocal cord tuberculosis and ankle tuberculosis were rarely reported; however, vocal cord tuberculosis with concurrent PTB was the strongest association rule in patients aged 45–54 years(lift = 1.61), while ankle joint tuberculosis with concurrent PTB was the strongest association rule in patients aged 55–64 years (lift = 1.55). Yet another form of EPTB, tuberculous pericarditis, refers to an M. tuberculosis infection of the membrane that covers the heart (pericardium). In endemic TB areas, tuberculous pericarditis has been found in 1% to 2% of people afflicted with pulmonary TB [26]. We found that tuberculous pericarditis with concurrent PTB was the strongest association rule in ≥65-year-old patients (lift = 1.42).

This study had several strengths, including its large-scale multicenter representative sample, its detailed analysis of diagnostic types of TB, and its novel determinations of confidence/lift values of concurrent PTB-EPTB cases. However, the study also had several limitations. First, it may have been subject to Berkson's bias since the study population included hospitalized TB patients of which concurrent PTB-EPTB cases had a high likelihood of being hospitalized causing the subsequent overestimation of their proportions within the general population. Therefore, data collected from whole population-based studies are still needed to clarify the associations. Secondly, most hospitals in our study were TB-specialized hospitals; thus, our findings may not represent the general TB patient population or apply to settings elsewhere in the country. Thirdly, some TB-specialized hospitals in China do not admit pediatric TB patients, and therefore, our results may not represent the general pediatric TB cases. Fourthly, TB diagnoses may be bacteriologically confirmed TB or clinically diagnosed TB. We selected the inpatients with confirmed TB diagnoses in the electronic patient medical databases. But we did not collect information on the number of bacteriologically or clinically diagnosed TB. Lastly, the aim of the study was to analyze the epidemiology and association rules analysis for pulmonary tuberculosis cases with extrapulmonary tuberculosis from age and gender perspectives, so the analysis did not consider low-frequency disease complications and comorbidities in China (e.g., HIV) and other factors such as new or retreatment, income, and smoking.

## 5. Conclusions

In conclusion, the present study revealed several types of concurrent PTB-EPTB cases and analyzed the association rules between PTB and EPTB for the first time in a large sample population. Concurrent PTB and tuberculous pleurisy was the most common concurrent PTB-EPTB case type observed. The strongest association rule in PTB with concurrent EPTB was PTB with concurrent bronchial tuberculosis. The strongest association rule in EPTB with concurrent PTB was pharyngeal/laryngeal tuberculosis with concurrent PTB. Confidence and lift values of concurrent PTB-EPTB cases varied with gender and age. Clinicians should be aware that concurrent PTB-EPTB cases are common and that these patients require the administration of customized treatment regimens in order to achieve the best outcomes.

## Figures and Tables

**Table 1:** The proportion of TB diagnosis (proportion ≥ 1.0%).

TB types	Frequency	Proportion (95% CI) (%)
Pulmonary tuberculosis	360187	82.0511 (81.9372–82.1645)
Tuberculous pleurisy	103680	23.6184 (23.4929–23.7444)
Bronchial tuberculosis	30779	7.0115 (6.9361–7.0874)
Tuberculous meningitis	15711	3.5790 (3.5242–3.6344)
Tuberculous lymphadenitis of the neck	15282	3.4813 (3.4272–3.5359)
Tuberculous peritonitis	10059	2.2915 (2.2474–2.3362)
Tuberculous empyema	7341	1.6723 (1.6346–1.7107)
Lumbar vertebra tuberculosis	7190	1.6379 (1.6006–1.6759)
Tuberculous pericarditis	5842	1.3308 (1.2971–1.3652)
Thoracic vertebra tuberculosis	5317	1.2112 (1.1791–1.2440)
Tuberculous polyserositis	4870	1.1094 (1.0786–1.1408)
Intestinal tuberculosis	4711	1.0732 (1.0429–1.1041)
Chest wall tuberculosis	4639	1.0568 (1.0267–1.0875)

*Note.* Proportion = frequency*∗*100%/438979.

**Table 2:** The associations between PTB concurrent with EPTB and gender, age group.

Characteristics	No. of PTB concurrent with EPTB (%)	aOR (95% CI)	*P*
Gender			
Female	49209 (31.7)	1.119 (1.104–1.134)	<0.001
Male	80213 (28.3)	Reference	

Age group(years)			
<15	2417 (34.2)	1.602 (1.521–1.686)	<0.001
15–24	27094 (37.2)	1.831 (1.792–1.871)	<0.001
25–34	26806 (35.6)	1.700 (1.664–1.738)	<0.001
35–44	17193 (30.5)	1.358 (1.327–1.391)	<0.001
45–54	18154 (25.8)	1.084 (1.059–1.109)	<0.001
55–64	16835 (23.7)	0.971 (0.949–0.994)	0.014
≥65	20923 (24.3)	Reference	

**Table 3:** The association rules of PTB with concurrent EPTB where antecedent = PTB, minconfidence = 1.00%.

Consequent	Antecedent	ID	Instances	Support (%)	Confidence (%)	Lift
Tuberculous pleurisy	Pulmonary tuberculosis	1	360187	82.05	18.71	0.79
Bronchial tuberculosis	Pulmonary tuberculosis	2	360187	82.05	7.65	1.09^*∗*^
Tuberculous meningitis	Pulmonary tuberculosis	3	360187	82.05	2.72	0.76
Tuberculous lymphadenitis of the neck	Pulmonary tuberculosis	4	360187	82.05	1.93	0.56
Tuberculous peritonitis	Pulmonary tuberculosis	5	360187	82.05	1.59	0.69
Tuberculous empyema	Pulmonary tuberculosis	6	360187	82.05	1.05	0.63

**Table 4:** The association rules of EPTB with concurrent PTB where consequent = PTB, minsupport = 0.1%, minconfidence = 40%.

Consequent	Antecedent	ID	Instances	Support (%)	Confidence (%)	Lift
PTB	Pharyngeal/laryngeal tuberculosis	1	2382	0.54	91.23	1.11^*∗*^
PTB	Bronchial tuberculosis	2	30779	7.01	89.51	1.09^*∗*^
PTB	Tuberculosis of mediastinal lymph nodes	3	3482	0.79	77.57	0.95
PTB	Tuberculosis of hilar lymph nodes	4	1117	0.25	74.93	0.91
PTB	Intestinal tuberculosis	5	4711	1.07	71.75	0.87
PTB	Tuberculosis of abdominal lymph nodes	6	651	0.15	66.36	0.81
PTB	Tuberculous pleurisy	7	103680	23.62	65.01	0.79
PTB	Tuberculous meningitis	8	15711	3.58	62.43	0.76
PTB	Tuberculous polyserositis	9	4870	1.11	59.22	0.72
PTB	Tuberculous pericarditis	10	5842	1.33	58.18	0.71
PTB	Tuberculous peritonitis	11	10059	2.29	56.99	0.69
PTB	Testicular tuberculosis	12	495	0.11	54.75	0.67
PTB	Tuberculous empyema	13	7341	1.67	51.53	0.63
PTB	Sacroiliac joint tuberculosis	14	574	0.13	50.87	0.62
PTB	Chest wall tuberculosis	15	4639	1.06	48.35	0.59
PTB	Thoracic vertebra tuberculosis	16	5317	1.21	46.00	0.56
PTB	Tuberculous lymphadenitis of the neck	17	15282	3.48	45.58	0.56
PTB	Pleural tuberculoma	18	917	0.21	45.37	0.55
PTB	Epididymal tuberculosis	19	794	0.18	45.21	0.55
PTB	Cutaneous tuberculosis	20	871	0.20	45.01	0.55
PTB	Renal tuberculosis	21	2793	0.64	43.04	0.52
PTB	Pelvic tuberculosis	22	1835	0.42	42.78	0.52

Notes: PTB: pulmonary tuberculosis. The first column represents the consequents (the “then” part of the rule), while the next column represents the antecedents (the “if” part of the rule). ID displays the sequence of the association rules. Instances display the cases of TB. ^*∗*^: lift>1.

## Data Availability

The datasets used or analyzed during the current study are available from the corresponding author upon reasonable request.

## Supplementary Materials

Supplementary Materials Supplementary Figure S1. The age-gender percent-bar-graph of patients with concurrent PTB-EPTB. Supplementary Tables S1. The TOP 20 most common PTB concurrent EPTB Table S2. The associations rules of concurrent PTB and EPTB in males (minsupport = 0.02%, minconfidence = 60%) Table S3. The association rules of concurrent PTB and EPTB in females (minsupport = 0.02%, minconfidence = 40%) Table S4. The association rules of concurrent PTB and EPTB in patients aged. Table S5. The association rules of concurrent PTB and EPTB in patients aged 15−24 years (minsupport = 0.02%, minconfidence = 20%) Table S6. The association rules of concurrent PTB and EPTB in patients aged 25–34 years (minsupport = 0.02%, minconfidence = 20%) Table S7. The association rules of concurrent PTB and EPTB in patients aged 35–44 years (minsupport = 0.02%, minconfidence = 15%) Table S8. The association rules of concurrent PTB and EPTB in patients aged 45–54 years (minsupport = 0.02%, minconfidence = 15%) Table S9. The association rules of concurrent PTB and EPTB in patients aged 55–64 years (minsupport = 0.02%, minconfidence = 15%) Table S10. The association rules of concurrent PTB and EPTB in patients aged ≥ 65 years (minsupport = 0.02%, minconfidence = 15%).
