# Supplementary material for: Epidemiology and Association Rules Analysis for Pulmonary Tuberculosis Cases with Extrapulmonary Tuberculosis from Age and Gender Perspective: A Large-Scale Retrospective Multicenter Observational Study in China
Source: Int J Clin Pract. 2023 Jul 31;2023:5562495. doi: 10.1155/2023/5562495 (PMC10442182; doi:10.1155/2023/5562495)
Supplement: Supplementary Materials — Supplementary Figure S1. The age-gender percent-bar-graph of patients with concurrent PTB-EPTB. Supplementary Tables S1. The TOP 20 most common PTB concurrent EPTB Table S2. The associations rules of concurrent PTB and EPTB in males (minsupport = 0.02%, minconfidence = 60%) Table S3. The association rules of concurrent PTB and EPTB in females (minsupport = 0.02%, minconfidence = 40%) Table S4. The association rules of concurrent PTB and EPTB in patients aged. Table S5. The association rules of concurrent PTB and EPTB in patients aged 15−24 years (minsupport = 0.02%, minconfidence = 20%) Table S6. The association rules of concurrent PTB and EPTB in patients aged 25–34 years (minsupport = 0.02%, minconfidence = 20%) Table S7. The association rules of concurrent PTB and EPTB in patients aged 35–44 years (minsupport = 0.02%, minconfidence = 15%) Table S8. The association rules of concurrent PTB and EPTB in patients aged 45–54 years (minsupport = 0.02%, minconfidence = 15%) Table S9. The association rules of concurrent PTB and EPTB in patients aged 55–64 years (minsupport = 0.02%, minconfidence = 15%) Table S10. The association rules of concurrent PTB and EPTB in patients aged ≥ 65 years (minsupport = 0.02%, minconfidence = 15%). [file 5562495.f1.pdf]

Supplementary Appendix

1.0 Supplementary Figure

2.0 Supplementary Tables

1.0 Supplementary Figure

Figure S1. The age-gender percent-bar-graph of patients with concurrent PTB-EPTB

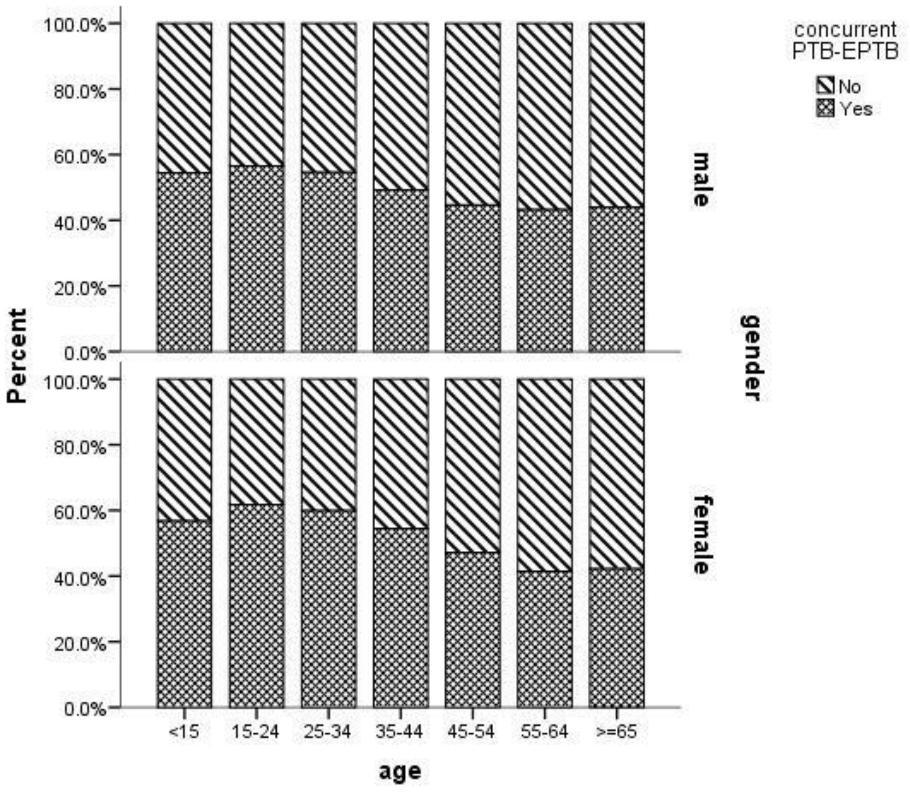

2.0 Supplementary Tables

Table S1. The TOP 20 most common of PTB concurrent EPTB

Table S2. The associations rules of concurrent PTB and EPTB in males (Minsupport = 0.02%, Minconfidence = 60%)

Table S3. The association rules of concurrent PTB and EPTB in females (Minsupport = 0.02%, Minconfidence = 40%)

Table S4. The association rules of concurrent PTB and EPTB in patients aged <15 years (Minsupport = 0.02%, Minconfidence = 3.0%)

Table S5. The association rules of concurrent PTB and EPTB in patients aged 15-24years (Minsupport = 0.02%, Minconfidence = 20%)

Table S6. The association rules of concurrent PTB and EPTB in patients aged 25-34 years (Minsupport = 0.02%, Minconfidence = 20%)

Table S7. The association rules of concurrent PTB and EPTB in patients aged 35-44 years (Minsupport = 0.02%, Minconfidence = 15%)

Table S8. The association rules of concurrent PTB and EPTB in patients aged 45-54 years (Minsupport = 0.02%, Minconfidence = 15%)

Table S9. The association rules of concurrent PTB and EPTB in patients aged 55-64 years (Minsupport = 0.02%, Minconfidence = 15%)

Table S10. The association rules of concurrent PTB and EPTB in patients aged ≥65 years (Minsupport = 0.02%, Minconfidence = 15%)

Table S1. The TOP 20 most common of PTB with concurrent EPTB

TOP	PTB with concurrent EPTB	Frequency	Proportion(95%CI) (%)
1	PTB with concurrent tuberculous pleurisy	67400	15.3538(15.2473-15.4608)
2	PTB with concurrent bronchial tuberculosis	27549	6.2757 (6.2041-6.3479)
3	PTB with concurrent tuberculous meningitis	9809	2.23 45(2.1910-2.2787)
4	PTB with concurrent tuberculous lymphadenitis of neck	6966	1.5869(1.5501-1.6243)
5	PTB with concurrent tuberculous peritonitis	5733	1.3060(1.2726-1.3400)
6	PTB with concurrent tuberculous empyema	3783	0.8618(0.8346-0.8896)
7	PTB with concurrent tuberculous pericarditis	3399	0.7743(0.7486-0.8007)
8	PTB with concurrent intestinal tuberculosis	3380	0.7700(0.7443-0.7963)
9	PTB with concurrent tuberculous polyserositis	2884	0.6570(0.6333-0.6813)
10	PTB with concurrent tuberculosis of mediastinal lymph nodes	2701	0.6153(0.5924-0.6389)
11	PTB with concurrent lumbar vertebra tuberculosis	2564	0.5841(0.5618-0.6071)
12	PTB with concurrent thoracic vertebra tuberculosis	2446	0.5572(0.5354-0.5797)
13	PTB with concurrent chest wall tuberculosis	2243	0.5110(0.4901-0.5325)
14	PTB with concurrent pharyngeal /laryngeal tuberculosis	2173	0.4950 (0.4745-0.5162)
15	PTB with concurrent renal tuberculosis	1202	0.2738(0.2586-0.2897)
16	PTB with concurrent tuberculosis of hilar lymph nodes	837	0.1907(0.1780-0.2040)
17	PTB with concurrent pelvic tuberculosis	785	0.1788 (0.1665-0.1918)
18	PTB with concurrent tuberculosis of axillary lymph nodes	593	0.1351(0.1244-0.1464)
19	PTB with concurrent knee joint tuberculosis	468	0.1066(0.0972-0.1167)
20	PTB with concurrent tuberculosis of abdominal lumph nodes	432	0.0 984(0.0894-0.1081)

%=frequency*100%/438979

The TOP 20 most common of concurrent EPTB and PTB were listed in table S1, sort by cases. The number of PTB concurrent with tuberculous pleurisy, bronchial tuberculosis, tuberculous meningitis, tuberculous lymphadenitis of neck and tuberculous peritonitis were all more than 5000 cases.

Table S2. The associations rules of concurrent PTB and EPTB in males (Minsupport = 0.02%, Minconfidence = 60%)

Consequent	Antecedent	ID	Instances	Support (%)	Confidence (%)	Lift
male	tuberculous empyema with concurrent PTB	1	3783	0.86	77.69	1.20*
male	costal tuberculosis with concurrent PTB	2	88	0.02	75.00	1.16*
male	tuberculous pleurisy with concurrent PTB	3	67400	15.35	70.63	1.09*
male	wrist joint tuberculosis with concurrent PTB	4	147	0.03	70.07	1.08*
male	adrenal tuberculosis with concurrent PTB	5	156	0.04	69.87	1.08*
male	hip joint tuberculosis with concurrent PTB	6	339	0.08	69.32	1.07*
male	tuberculosis of abdominal lumph nodes with concurrent PTB	7	432	0.10	66.67	1.03*
male	tuberculous abscess of psoas major with concurrent PTB	8	169	0.04	66.27	1.02*
male	intestinal tuberculosis with concurrent PTB	9	3380	0.77	66.07	1.02*
male	knee joint tuberculosis with concurrent PTB	10	468	0.11	66.03	1.02*
male	pharyngeal /laryngeal tuberculosis with concurrent PTB	11	2173	0.50	65.21	1.01*
male	vocal cord tuberculosis with concurrent PTB	12	97	0.02	64.95	1.00
male	chest wall tuberculosis with concurrent PTB	13	2243	0.51	64.11	0.99
male	tuberculous pericarditis with concurrent PTB	14	3399	0.77	62.90	0.97
male	nasal tuberculosis with concurrent PTB	15	107	0.02	62.62	0.97
male	cervical vertebra tuberculosis with concurrent PTB	16	155	0.04	62.58	0.97
male	lumbar vertebra tuberculosis with concurrent PTB	17	2564	0.58	62.56	0.97
male	thoracic vertebra tuberculosis with concurrent PTB	18	2446	0.56	62.31	0.96
male	tuberculous polyserositis with concurrent PTB	19	2884	0.66	62.10	0.96

male	tuberculous myelitis with concurrent PTB	20	163	0.04	61.96	0.96
male	brain tuberculoma with concurrent PTB	21	203	0.05	61.58	0.95
male	elbow joint tuberculosis with concurrent PTB	22	97	0.02	60.82	0.94
male	pleural tuberculoma with concurrent PTB	23	416	0.09	60.58	0.94

In order to find out most of possible association rules in male, the minimum support degree was set as 0.02%, and the minimum confidence degree was set as 60.0%.After executing the association model, 23 association rules, including 4 rules whose confidence degree greater than 70% were obtained. In males, tuberculous empyema with concurrent PTB (77.69%) was the strongest association rule, followed by costal tuberculosis with concurrent PTB, tuberculous pleurisy with concurrent PTB, etc.

Table S3. The association rules of concurrent PTB and EPTB in females (Minsupport = 0.02%, Minconfidence = 40%)

Consequent	Antecedent	ID	Instances	Support (%)	Confidence (%)	Lift
female	bronchial tuberculosis with concurrent PTB	1	27549	6.28	57.77	1.64*
female	supraclavicular lymph node tuberculosis with concurrent PTB	2	169	0.04	55.03	1.56*
female	cutaneous tuberculosis with concurrent PTB	3	392	0.09	54.85	1.55*
female	ureteric tuberculosis with concurrent PTB	4	92	0.02	54.35	1.54*
female	tuberculosis of axillary lymph nodes concurrent PTB	5	593	0.14	52.28	1.48*
female	submaxillary lymph node tuberculosis with concurrent PTB	6	123	0.03	51.22	1.45*
female	tuberculous lymphadenitis of neck with concurrent PTB	7	6966	1.59	50.78	1.44*
female	hepatic tuberculosis with concurrent PTB	8	244	0.06	47.95	1.36*
female	sacral tuberculosis with concurrent PTB	9	95	0.02	45.26	1.28*
female	sacroiliac joint tuberculosis with concurrent PTB	10	292	0.07	44.86	1.27*
female	tuberculosis of hilar lymph nodes concurrent PTB	11	837	0.19	44.80	1.27*
female	renal tuberculosis with concurrent PTB	12	1202	0.27	44.34	1.25*
female	shoulder joint tuberculosis with concurrent PTB	13	103	0.02	42.72	1.21*
female	splenic tuberculosis with concurrent PTB	14	260	0.06	42.31	1.20*
female	abdominal wall tuberculosis with concurrent PTB	15	143	0.03	41.96	1.19*
female	tuberculosis of mediastinal lymph nodes with concurrent PTB	16	2701	0.62	41.76	1.18*
female	ankle joint tuberculosis with concurrent PTB	17	104	0.02	41.35	1.17*
female	inguinal lymph node tuberculosis with concurrent	18	119	0.03	41.18	1.17*

	PTB					
female	tuberculous peritonitis with concurrent PTB	19	5733	1.31	41.11	1.16*
female	tuberculous meningitis with concurrent PTB	20	9809	2.23	40.59	1.15*

In order to find out most of possible association rules in female, the minimum support degree was set as 0.02%, and the minimum confidence degree was set as 40.0%.After executing the association model, 20 association rules, including 2 rules whose confidence degree greater than 55% were obtained. In females, bronchial tuberculosis with concurrent PTB was the strongest association rule, followed by supraclavicular lymph node tuberculosis with concurrent PTB,cutaneous tuberculosis with concurrent PTB, etc.

Table S4. The association rules of concurrent PTB and EPTB in patients aged <15 years (Minsupport = 0.02%, Minconfidence = 3.0%)

Consequent	Antecedent	ID	Instances	Support (%)	Confidence (%)	Lift
age <15	tuberculous meningitis with concurrent PTB	1	9809	2.23	6.26	3.89*
age <15	tuberculosis of axillary lymph nodes with concurrent PTB	2	593	0.14	6.07	3.78*
age <15	cervical vertebra tuberculosis with concurrent PTB	3	155	0.04	5.81	3.61*
age <15	hip joint tuberculosis with concurrent PTB	4	339	0.08	5.31	3.30*
age <15	tuberculosis of hilar lymph nodes with concurrent PTB	5	837	0.19	4.90	3.05*
age <15	hepatic tuberculosis with concurrent PTB	6	244	0.06	4.10	2.55*
age <15	tuberculosis of mediastinal lymph nodes with concurrent PTB	7	2701	0.62	3.55	2.21*
age <15	abdominal wall tuberculosis with concurrent PTB	8	143	0.03	3.50	2.17*
age <15	inguinal lymph node tuberculosis with concurrent PTB	9	119	0.03	3.36	2.09*
age <15	tuberculous lymphadenitis of neck with concurrent PTB	10	6966	1.59	3.36	2.09*
age <15	tuberculous myelitis with concurrent PTB	11	163	0.04	3.07	1.91*

In order to find out most of possible concurrent PTB and EPTB association rules in age <15 years, the minimum support degree was set as 0.02%, and the minimum confidence degree was set as 3.0%.After executing the association model, 11 association rules were obtained.In patients <15 years of age, tuberculous meningitis with concurrent PTB was the strongest association rule, followed by tuberculosis of axillary lymph nodes with concurrent PTB, etc.

Table S5. The association rules of concurrent PTB and EPTB in patients aged 15-24years (Minsupport = 0.02%, Minconfidence = 20%)

Consequent	Antecedent	ID	Instances	Support (%)	Confidence (%)	Lift
age = 15-24	splenic tuberculosis with concurrent PTB	1	260	0.06	36.92	2.23*
age = 15-24	tuberculous myelitis with concurrent PTB	2	163	0.04	36.20	2.18*
age = 15-24	tuberculous peritonitis with concurrent PTB	3	5733	1.31	32.04	1.93*
age = 15-24	tuberculosis of abdominal lymph nodes with concurrent PTB	4	432	0.10	31.48	1.90*
age = 15-24	pelvic tuberculosis with concurrent PTB	5	785	0.18	29.81	1.80*
age = 15-24	brain tuberculoma with concurrent PTB	6	203	0.05	29.56	1.78*
age = 15-24	hepatic tuberculosis with concurrent PTB	7	244	0.06	29.51	1.78*
age = 15-24	tuberculous lymphadenitis of neck with concurrent PTB	8	6966	1.59	29.33	1.77*
age = 15-24	submaxillary lymph node tuberculosis with concurrent PTB	9	123	0.03	29.27	1.76*
age = 15-24	abdominal wall tuberculosis with concurrent PTB	10	143	0.03	28.67	1.73*
age = 15-24	tuberculous abscess of psoas major with concurrent PTB	11	169	0.04	28.40	1.71*
age = 15-24	pleural tuberculoma with concurrent PTB	12	416	0.09	27.64	1.67*
age = 15-24	intestinal tuberculosis with concurrent PTB	13	3380	0.77	27.60	1.66*
age = 15-24	chest wall tuberculosis with concurrent PTB	14	2243	0.51	27.42	1.65*
age = 15-24	sacral tuberculosis with concurrent PTB	15	95	0.02	27.37	1.65*
age = 15-24	sacroiliac joint tuberculosis with concurrent PTB	16	292	0.07	26.71	1.61*
age = 15-24	tuberculous polyserositis with concurrent PTB	17	2884	0.66	26.49	1.60*
age = 15-24	tuberculous meningitis with concurrent PTB	18	9809	2.23	26.22	1.58*
age = 15-24	tuberculous empyema with concurrent PTB	19	3783	0.86	26.20	1.58*
age = 15-24	tuberculosis of axillary lymph nodes with concurrent PTB	20	593	0.14	23.95	1.44*
age = 15-24	nasal tuberculosis with concurrent PTB	21	107	0.02	23.36	1.41*
age = 15-24	tuberculosis of mediastinal lymph nodes with concurrent PTB	22	2701	0.62	22.29	1.34*

age = 15-24	supraclavicular lymph node tuberculosis with concurrent PTB	23	169	0.04	21.89	1.32*
age = 15-24	inguinal lymph node tuberculosis with concurrent PTB	24	119	0.03	21.85	1.32*
age = 15-24	tuberculous pleurisy with concurrent PTB	25	67400	15.35	20.10	1.21*

In order to find out most of possible concurrent PTB and EPTB association rules in age 15-24 years, the minimum support degree was set as 0.02%, and the minimum confidence degree was set as 20.0%.After executing the association model, 25 association rules, including 4 rules whose confidence degree greater than 30% were obtained. In patients aged 15-24 years, splenic tuberculosis with concurrent PTB was the strongest association rule, followed by tuberculous myelitis with concurrent PTB, tuberculous peritonitis with concurrent PTB, etc.

Table S6. The association rules of concurrent PTB and EPTB in patients aged 25-34 years (Minsupport = 0.02%, Minconfidence = 20%)

Consequent	Antecedent	ID	Instances	Support (%)	Confidence (%)	Lift
age = 25-34	oviduct tuberculosis with concurrent PTB	1	100	0.02	53.00	3.09*
age = 25-34	endometrial tuberculosis with concurrent PTB	2	129	0.03	37.21	2.17*
age = 25-34	pelvic tuberculosis with concurrent PTB	3	785	0.18	36.82	2.15*
age = 25-34	tuberculous lymphadenitis of neck with concurrent PTB	4	6966	1.59	29.26	1.71*
age = 25-34	submaxillary lymph node tuberculosis with concurrent PTB	5	123	0.03	26.83	1.56*
age = 25-34	cutaneous tuberculosis with concurrent PTB	6	392	0.09	25.51	1.49*
age = 25-34	sacroiliac joint tuberculosis with concurrent PTB	7	292	0.07	25.34	1.48*
age = 25-34	pleural tuberculoma with concurrent PTB	8	416	0.09	25.24	1.47*
age = 25-34	supraclavicular lymph node tuberculosis with concurrent PTB	9	169	0.04	24.85	1.45*
age = 25-34	bronchial tuberculosis with concurrent PTB	10	27549	6.28	24.56	1.43*
age = 25-34	nasal tuberculosis with concurrent PTB	11	107	0.02	24.30	1.42*
age = 25-34	tuberculosis of abdominal lumph nodes with concurrent PTB	12	432	0.10	23.38	1.36*
age = 25-34	tuberculous abscess of psoas major with concurrent PTB	13	169	0.04	23.08	1.35*
age = 25-34	hepatic tuberculosis with concurrent PTB	14	244	0.06	22.95	1.34*
age = 25-34	chest wall tuberculosis with concurrent PTB	15	2243	0.51	22.87	1.33*
age = 25-34	ureteric tuberculosis with concurrent PTB	16	92	0.02	22.83	1.33*
age = 25-34	costal tuberculosis with concurrent PTB	17	88	0.02	22.73	1.33*
age = 25-34	inguinal lymph node tuberculosis with concurrent PTB	18	119	0.03	22.69	1.32*
age = 25-34	pharyngeal/Laryngeal tuberculosis with concurrent PTB	19	2173	0.50	22.60	1.32*
age = 25-34	tuberculosis of axillary lymph nodes with concurrent PTB	20	593	0.14	22.43	1.31*
age = 25-34	intestinal Tuberculosis with concurrent PTB	21	3380	0.77	22.28	1.30*
age = 25-34	tuberculous peritonitis with concurrent PTB	22	5733	1.31	22.19	1.29*

age = 25-34	splenic tuberculosis with concurrent PTB	23	260	0.06	21.15	1.23*
age = 25-34	abdominal wall tuberculosis with concurrent PTB	24	143	0.03	20.98	1.22*
age = 25-34	tuberculous myelitis with concurrent PTB	25	163	0.04	20.86	1.22*
age = 25-34	tuberculous meningitis with concurrent PTB	26	9809	2.23	20.66	1.20*
age = 25-34	elbow joint tuberculosis with concurrent PTB	27	97	0.02	20.62	1.20*
age = 25-34	lumbar vertebra tuberculosis with concurrent PTB	28	2564	0.58	20.28	1.18*

In order to find out most of possible concurrent PTB and EPTB association rules in age 25-34 years, the minimum support degree was set as 0.02%, and the minimum confidence degree was set as 20.0%.After executing the association model, 28 association rules, including 3 rules whose confidence degree greater than 30% were obtained. In patients aged 25-34, oviduct tuberculosis with concurrent PTB was the strongest association rule, followed by endometrial tuberculosis with concurrent PTB, etc.

Table S7. The association rules of concurrent PTB and EPTB in patients aged 35-44 years (Minsupport = 0.02%, Minconfidence = 15%)

Consequent	Antecedent	ID	Instances	Support (%)	Confidence (%)	Lift
age = 35-44	oviduct tuberculosis with concurrent PTB	1	100	0.02	22.00	1.71*
age = 35-44	endometrial tuberculosis with concurrent PTB	2	129	0.03	21.71	1.69*
age = 35-44	ureteric tuberculosis with concurrent PTB	3	92	0.02	19.57	1.52*
age = 35-44	epididymal tuberculosis with concurrent PTB	4	359	0.08	19.22	1.50*
age = 35-44	testicular tuberculosis with concurrent PTB	5	271	0.06	19.19	1.50*
age = 35-44	knee joint tuberculosis with concurrent PTB	6	468	0.11	19.02	1.48*
age = 35-44	ankle joint tuberculosis with concurrent PTB	7	104	0.02	18.27	1.42*
age = 35-44	brain tuberculoma with concurrent PTB	8	203	0.05	18.23	1.42*
age = 35-44	adrenal tuberculosis with concurrent PTB	9	156	0.04	17.95	1.40*
age = 35-44	inguinal lymph node tuberculosis with concurrent PTB	10	119	0.03	17.65	1.38*
age = 35-44	pelvic tuberculosis with concurrent PTB	11	785	0.18	16.18	1.26*
age = 35-44	pharyngeal/laryngeal tuberculosis with concurrent PTB	12	2173	0.50	16.01	1.25*
age = 35-44	supraclavicular lymph node tuberculosis with concurrent PTB	13	169	0.04	15.98	1.24*
age = 35-44	vocal cord tuberculosis with concurrent PTB	14	97	0.02	15.46	1.21*
age = 35-44	bronchial tuberculosis with concurrent PTB	15	27549	6.28	15.20	1.18*
age = 35-44	cutaneous tuberculosis with concurrent PTB	16	392	0.09	15.05	1.17*

In order to find out most of possible concurrent PTB and EPTB association rules in age 35-44 years, the minimum support degree was set as 0.02%, and the minimum confidence degree was set as 15.0%.After executing the association model, 16 association rules, including 2 rules whose confidence degree greater than 20% were obtained. In patients aged 35-44 years, the strongest association rule was oviduct tuberculosis with concurrent PTB, followed by endometrial tuberculosis with concurrent PTB, etc.

Table S8. The association rules of concurrent PTB and EPTB in patients aged 45-54 years (Minsupport = 0.02%, Minconfidence = 15%)

Consequent	Antecedent	ID	Instances	Support (%)	Confidence (%)	Lift
age = 45-54	vocal cord tuberculosis with concurrent PTB	1	97	0.02	25.77	1.61*
age = 45-54	wrist joint tuberculosis with concurrent PTB	2	147	0.03	25.17	1.57*
age = 45-54	adrenal tuberculosis with concurrent PTB	3	156	0.04	25.00	1.56*
age = 45-54	ureteric tuberculosis with concurrent PTB	4	92	0.02	22.83	1.42*
age = 45-54	nasal tuberculosis with concurrent PTB	5	107	0.02	19.63	1.22*
age = 45-54	renal tuberculosis with concurrent PTB	6	1202	0.27	19.38	1.21*
age = 45-54	epididymal tuberculosis with concurrent PTB	7	359	0.08	18.66	1.16*
age = 45-54	elbow joint tuberculosis with concurrent PTB	8	97	0.02	18.56	1.16*
age = 45-54	hip joint tuberculosis with concurrent PTB	9	339	0.08	17.99	1.12*
age = 45-54	pharyngeal/laryngeal tuberculosis with concurrent PTB	10	2173	0.50	17.76	1.11*
age = 45-54	knee joint tuberculosis with concurrent PTB	11	468	0.11	17.74	1.11*
age = 45-54	testicular tuberculosis with concurrent PTB	12	271	0.06	17.34	1.08*
age = 45-54	abdominal wall tuberculosis with concurrent PTB	13	143	0.03	16.78	1.05*
age = 45-54	tuberculous empyema with concurrent PTB	14	3783	0.86	15.99	1.00*
age = 45-54	sacroiliac joint tuberculosis with concurrent PTB	15	292	0.07	15.07	0.94

In order to find out most of possible concurrent PTB and EPTB association rules in age 45-54 years, the minimum support degree was set as 0.02%, and the minimum confidence degree was set as 15.0%.After executing the association model, 15 association rules, including 4 rules whose confidence degree greater than 20% were obtained. In patients aged 45-54 years, the strongest association rule was vocal cord tuberculosis with concurrent PTB, followed by wrist joint tuberculosis with concurrent PTB, etc.

Table S9. The association rules of concurrent PTB and EPTB in patients aged 55-64 years (Minsupport = 0.02%, Minconfidence = 15%)

Consequent	Antecedent	ID	Instances	Support (%)	Confidence (%)	Lift
age = 55-64	ankle joint tuberculosis with concurrent PTB	1	104	0.02	25.00	1.55*
age = 55-64	adrenal tuberculosis with concurrent PTB	2	156	0.04	24.36	1.51*
age = 55-64	shoulder joint tuberculosis with concurrent PTB	3	103	0.02	22.33	1.38*
age = 55-64	vocal cord tuberculosis with concurrent PTB	4	97	0.02	20.62	1.28*
age = 55-64	testicular tuberculosis with concurrent PTB	5	271	0.06	18.45	1.14*
age = 55-64	hip joint tuberculosis with concurrent PTB	6	339	0.08	18.29	1.13*
age = 55-64	tuberculosis of hilar lymph nodes with concurrent PTB	7	837	0.19	17.92	1.11*
age = 55-64	knee joint tuberculosis with concurrent PTB	8	468	0.11	17.74	1.10*
age = 55-64	cervical vertebra tuberculosis with concurrent PTB	9	155	0.04	17.42	1.08*
age = 55-64	renal tuberculosis with concurrent PTB	10	1202	0.27	16.64	1.03*
age = 55-64	thoracic vertebra tuberculosis with concurrent PTB	11	2446	0.56	16.52	1.02*
age = 55-64	tuberculous pericarditis with concurrent PTB	12	3399	0.77	16.45	1.02*
age = 55-64	wrist joint tuberculosis with concurrent PTB	13	147	0.03	15.65	0.97
age = 55-64	tuberculosis of mediastinal lymph nodes with concurrent PTB	14	2701	0.62	15.55	0.96

In order to find out most of possible concurrent PTB and EPTB association rules in age 55-64 years, the minimum support degree was set as 0.02%, and the minimum confidence degree was set as 15.0%.After executing the association model, 14 association rules, including 4 rules whose confidence degree greater than 20% were obtained. In TB patients aged 55-64 years, the strongest association rule was ankle joint tuberculosis with concurrent PTB, followed by adrenal tuberculosis with concurrent PTB, etc.

Table S10. The association rules of concurrent PTB and EPTB in patients aged ≥65 years (Minsupport = 0.02%, Minconfidence = 15%)

Consequent	Antecedent	ID	Instances	Support (%)	Confidence (%)	Lift
age ≥65	tuberculous pericarditis with concurrent PTB	1	3399	0.77	27.95	1.42*
age ≥65	tuberculosis of hilar lymph nodes with concurrent PTB	2	837	0.19	25.45	1.30*
age ≥65	shoulder joint tuberculosis with concurrent PTB	3	103	0.02	25.24	1.29*
age ≥65	cervical vertebra tuberculosis with concurrent PTB	4	155	0.04	22.58	1.15*
age ≥65	thoracic vertebra tuberculosis with concurrent PTB	5	2446	0.56	21.10	1.07*
age ≥65	ankle joint tuberculosis with concurrent PTB	6	104	0.02	20.19	1.03*
age ≥65	lumbar vertebra tuberculosis with concurrent PTB	7	2564	0.58	19.73	1.01*
age ≥65	wrist joint tuberculosis with concurrent PTB	8	147	0.03	19.73	1.00*
age ≥65	tuberculous pleurisy with concurrent PTB	9	67400	15.35	19.60	1.00*
age ≥65	elbow joint tuberculosis with concurrent PTB	10	97	0.02	19.59	1.00*
age ≥65	mediastinal lymph node tuberculosis with concurrent PTB	11	2701	0.62	18.92	0.96
age ≥65	costal tuberculosis with concurrent PTB	12	88	0.02	18.18	0.93
age ≥65	tuberculous polyserositis with concurrent PTB	13	2884	0.66	17.79	0.91
age ≥65	sacral tuberculosis with concurrent PTB	14	95	0.02	16.84	0.86
age ≥65	knee joint tuberculosis with concurrent PTB	15	468	0.11	16.24	0.83

In order to find out most of possible concurrent PTB and EPTB association rules in ≥65 years, the minimum support degree was set as 0.02%, and the minimum confidence degree was set as 15.0%.After executing the association model, 15 association rules, including 6 rules whose confidence degree greater than 20% were obtained. In TB patients aged ≥65 years,

the strongest association rule was tuberculous pericarditis with concurrent PTB, followed by hilar lymph nodes with concurrent PTB, etc.

Notes: The first column represents the consequents (the "then" part of the rule), while the next column represents the antecedents (the "if" part of the rule).
ID displays the sequence of the association rules.
Instances display the cases of TB.
*：lift⩾1
